# Characteristics of contrast‐enhanced ultrasound for diagnosis of solid clear cell renal cell carcinomas ≤4 cm: A meta‐analysis

**DOI:** 10.1002/cam4.4365

**Published:** 2021-11-01

**Authors:** Yang Liu, Yanmin Kan, Jincun Zhang, Ning Li, Yihua Wang

**Affiliations:** ^1^ Department of Ultrasound The Affiliated Hospital of North China University of Science and Technology Tangshan Hebei P.R. China; ^2^ Department of Ultrasound Tianjin Third Central Hospital Tianjin P.R. China; ^3^ Department of Urological Surgery The Affiliated Hospital of North China University of Science and Technology Tangshan Hebei P.R. China

**Keywords:** clear cell renal cell carcinoma, contrast‐enhanced ultrasound, meta‐analysis, solid tumor, tumor characteristics

## Abstract

Now solid renal tumors ≤4 cm is the most common, especially the subtype of clear cell renal cell carcinoma (ccRCC) of malignant kidney tumors in clinical. However, there is not specific characteristics of contrast‐enhanced ultrasound (CEUS) be recommended by the EFSUMB Guidelines in distinguish the essence of the kidney tumor with different sizes. Therefore, this meta‐analysis aimed to assess the ability of CEUS to diagnose solid ccRCC (sccRCC) ≤4 cm. We comprehensively searched the Cochrane Library, Embase, PubMed, and Web of Science databases from their inception to 28 July 2020, for studies reporting the CEUS features of sccRCC lesions ≤4 cm. Additional articles were identified through the Chinese National Knowledge Infrastructure database. Studies were selected independently by two investigators and the relevant data were extracted. Discrepancies were resolved via discussion with the senior author. Study quality was assessed using the Quality Assessment of Diagnostic Accuracy Studies‐2 tool, and the sensitivity and specificity of each study were determined and plotted as a receiver operating characteristic curve. Ten studies were included in this meta‐analysis. Hyperenhancement showed medium sensitivity (67%–89%) and specificity (42%–75%) for diagnosing sccRCC ≤4 cm, fast‐in contrast agent and heterogeneous enhancement showed high diagnostic abilities (area under curve (AUC) 0.74–0.84), but the presence of a pseudocapsule and fast‐out contrast agent had poor diagnostic ability (AUC <0.70). The combination of hyperenhancement and iso‐enhancement showed high sensitivity (98%) for diagnosing sccRCC ≤4 cm. Hyperenhancement, fast‐in contrast agent, and heterogeneous enhancement may be specific features that could help to identify sccRCC ≤4 cm, while the presence of a pseudocapsule and fast‐out of contrast agent may have low diagnostic values. The combination of multiple indexes may improve the diagnostic value of CEUS for sccRCC ≤4 cm.

## Graphical abstract

Now solid renal tumors ≤4 cm is the most common, especially the subtype of clear cell renal cell carcinoma (ccRCC) of malignant kidney tumors in clinical. However, there is not specific characteristics of contrast‐enhanced ultrasound (CEUS) with the EFSUMB Guidelines be recommended in distinguish the essence of the kidney tumor with different sizes. Therefore, this meta‐analysis aimed to assess the ability of CEUS to diagnose solid ccRCC (sccRCC) ≤4 cm.

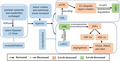

## 1 INTRODUCTION

Renal cell carcinoma (RCC) is a highly vascularized malignant tumor originating from the urinary tubular epithelium of the renal parenchyma, accounting for 80%–90% of renal malignancies.^1^ The incidence of RCC is steadily increasing at a rate of 3.7% per year,^2^ it was estimated that there were 403,262 new cases and 175,098 deaths in 185 countries according to global cancer statistics 2018, people's health is seriously threatened by RCC. The detection rate of small renal masses is gradually increasing because the use of image technology has boosted. It was defined as a tumor equal or less than 4 cm in maximum axial diameter, in which about 80% were malignant, and most of them were small renal carcinoma.^3^ Different RCC subtypes have been identified based on primary tumor histology, morphology, and cytogenetics, with ccRCC being the most common subtype, accounting for about 70%–80% of all renal tumors, and the 5‐year overall survival rate of early stage ccRCC could reach 96%, but it is no more than 10% for advanced stages.^4^ Correspondingly, majority of small renal carcinoma are at early stage. Therefore, the early detection and timely treatment for sccRCC ≤4 cm become one of the most elements in improving curative effect and survival status.

Renal diseases are usually examined by computed tomography (CT), magnetic resonance imaging (MRI), conventional ultrasound (CUS), and CEUS. CT and MRI have high diagnostic capability in detecting renal tumor, but they do not apply to all the crowd for ionizing radiation (CT) or exorbitant fees (MRI). CUS provides a fast, safe, repeatable, and cost‐effective method for revealing the basic characteristics of lesions, such as location, size, shape, border, echo, and blood supply. However, it does not recognize tumor microflow and detection rate of small renal tumors is lower than CT, especially those less than 2 cm.^3^ CEUS has been developed and is now widely used for safe and dynamic real‐time imaging; the injection of a contrast agent can increase the contrast between tissues and highlight subtle differences in blood flow signals in the lesion, thus helping to determine the nature of the lesion or its subtype; and CEUS has recently been increasingly applied to differentiate small renal masses. However, the performance of RCC by CEUS might be influenced by pathological subtypes and sizes.^5^ In conventional ultrasound and CEUS, there are overlap manifestations of echo, enhancement degree, uniformity, and the appearance of the pseudocapsule with ≤4 cm renal tumors. But the EFSUMB Guidelines only proposed that CEUS was used to diagnosis solid renal masses, not to mention specific characteristics of CEUS. ^6^ In addition, few studies have examined CEUS findings in sccRCC ≤4 cm. This meta‐analysis aimed to review the CEUS features of sccRCC lesions ≤4 cm to explore their utility for diagnosing sccRCC ≤4 cm.

## 2 MATERIALS AND METHODS

The study did not require approval from the institutional Research Ethics Board. The study protocol has been published in PROSPERO (International Prospective Register of Systematic Reviews; registration number: CRD42021237416; 19 March 2021). The study was conducted in accordance with the QUORUM reporting guidelines.

The study addressed the following issues, according to the “PICOS” framework: P (patients): adults (age >18 years) with a solid renal mass ≤4 cm, possibly representing a ccRCC^7^, ^8^; I (intervention): CEUS (implemented before clinical intervention); C (comparison): no comparison; O (outcome): reference standard of histopathological confirmation from surgery or tissue biopsy; and S (study type): any study that included ≥30 cases.^9^

### 2.1 Literature search

A literature search was performed using the Embase, PubMed, Web of Science, and Cochrane databases from their inception to 28 July 2020, with no language restrictions. The keywords included: “clear cell renal cell carcinoma,” “ccRCC,” “renal clear cell carcinoma,” “contrast‐enhanced ultrasound,” “contrast‐enhanced ultrasonography,” “contrast ultrasonography,” “ultrasound contrast imaging,” and “CEUS.” Additional studies were identified through the Chinese National Knowledge Infrastructure database. Titles and abstracts were reviewed and eligibility was determined independently by two investigators with disagreements resolved by consensus, and full‐text versions of relevant studies were retrieved for further evaluation.

### 2.2 Inclusion and exclusion criteria

The inclusion criteria were as follows: (1) adults with sccRCC ≤4 cm; (2) CEUS implemented before clinical intervention; (3) data retrievable to calculate a 2 × 2 contingency table; (4) acceptable reference standard (pathology) used for all patients; (5) CEUS characteristics of the mass described qualitatively or quantitatively; and (6) study patients not a subset of patients from another included paper. In the event of overlap between study samples, the latest results were used.

The exclusion criteria were as follows: (1) studies with fewer than 30 cases; and (2) conference papers or secondary literature, such as experience exchanges, abstracts, lectures, and reviews.

### 2.3 Data extraction

All retrieved articles were exported into Endnote 9.3.3, and all duplicate studies were removed. Each article was assessed independently by two investigators, and eligible studies were recruited according to the selection criteria. The investigators extracted the relevant data into a data extraction table (Microsoft Excel), and any discrepancies were discussed with a third reviewer to reach an agreement. The following data were gathered: first author's surname, year of publication, geographical location, language, ethnicity, sex, age, number of sccRCCs and lesions other than sccRCCs ≤4 cm, lesion length, study design, CEUS instrument, contrast agent type and dose, mechanical index, “gold standard,” true positive (TP), true negative (TN), false positive (FP), and false negative (FN) CEUS characteristics. The definitions were as follows: (1) hyper‐, iso‐, and hypo‐enhancement at peak lesion enhancement were defined according to the lesion‐enhancement degree compared with the renal cortex; (2) “fast‐in” and “slow‐in” indicated an arrival time of the contrast agent in the lesion before or after its arrival in the adjacent renal cortex, and “fast‐out” and “slow‐out” indicated the relative outflow of the agent from the tumor, respectively; (3) homogenous enhancement was defined as a lesion with full enhancement without any defects, and heterogeneous enhancement was defined as a lesion with some unenhanced areas; (4) a rim of perilesional enhancement was considered to represent the pseudocapsule; (5) TP and FN were CEUS characteristics of sccRCC ≤4 cm, TP was present, FN was absent; and (6) FP and TN were CEUS characteristics of lesions other than sccRCC ≤4 cm, FP was present, TN was absent.

### 2.4 Assessment of quality

The Quality Assessment of Diagnostic Accuracy Studies‐2 (QUADAS‐2) tool^7^ was used to evaluate the risk of bias and methodological quality by two investigators, independently. This tool consisted of two main domains: risk of bias, which in turn included patient selection, index test, reference standard, and flow and timing, with each item judged as low, high, or unclear risk; and applicability concerns, which included patient selection, index test, and reference standard, with each item considered as low, high, or unclear concern. If any of these items was identified as high risk or high concern, the study was judged to be at high risk of bias.^8^

### 2.5 Statistical analysis

All data were calculated using STATA 15.1 and Review Manager 5.3 software. Quality assessment was performed using Review Manager 5.3. The following indexes were calculated to assess the diagnostic ability of the CEUS characteristics: sensitivity, specificity, diagnostic odds ratio (DOR), negative likelihood ratio (NLR), positive likelihood ratio (PLR), area under the curve (AUC) with 95% confidence intervals (CIs), summary receiver operating characteristic curve (SROC), and percentage of patients with the CEUS characteristics. The 2 × 2 data were summarized in forest plots of sensitivity and specificity for each CEUS characteristic. Sensitivity and specificity on a per‐feature basis were plotted in SROC space. Cochran's Q‐statistic and *I* ^2^ tests were used to evaluate the potential heterogeneity between studies. *I* ≤25% indicated low heterogeneity, >25% to ≤50% indicated mild heterogeneity, >50% to ≤75% indicated moderate heterogeneity, and *I* ^2^ > 75% indicated high heterogeneity. If *I* ^2^ > 50%, a random‐effect model was used.^10^ We also performed sub‐group analyses based on language, ethnicity, lesion length, and contrast agent type, to investigate potential sources of heterogeneity. We evaluated the influence of each study on the overall estimate by sensitivity analysis. Reporting bias was checked by funnel plots, Begg’s test, and Egger’s test. *p* < 0.05 indicated statistical significance.

## 3 RESULTS

### 3.1 Baseline characteristics of the included studies

**FIGURE 1:**
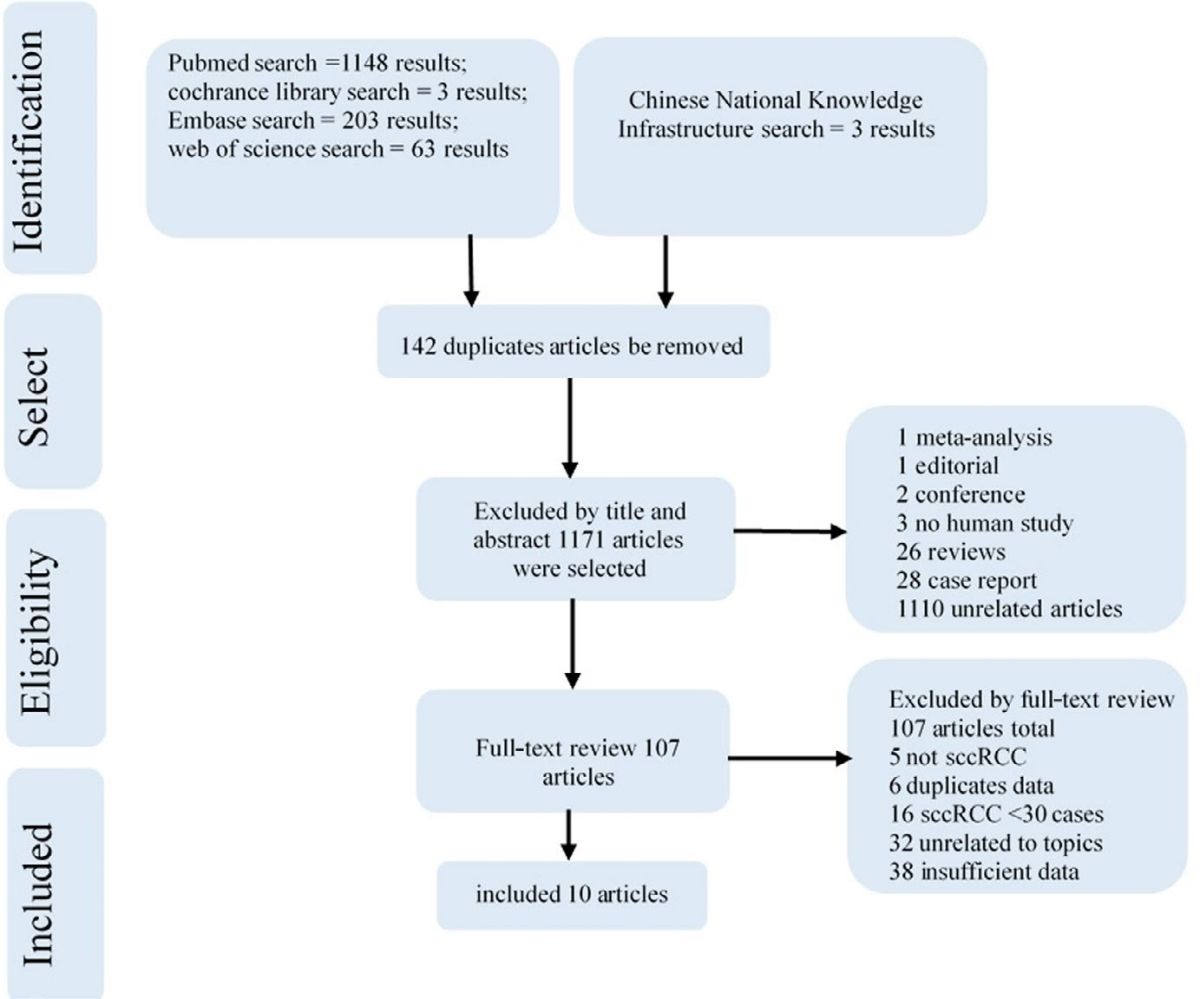
Flow diagram showing the article selection procedure. Ten articles were finally included in this meta‐analysis to calculate the percentage of CEUS characteristics, and nine articles were included for qualitative analysis of CEUS characteristics

A flow diagram of the study selection procedure is presented in Figure 1. Finally, 10 articles were included in the meta‐analyses. The characteristics of the 10 included articles are listed in Table 1. All of the studies were retrospective, and the publication dates ranged from 2010 to 2019. Five studies included lesions ≤4 cm (255 sccRCC, 116 other lesions) and five included lesions ≤3 cm (342 sccRCC, 114 other lesions). Eight studies included patients from Asia, and the remaining two study populations were from Canada, and Iksan, Korea, respectively. The contrast agent definity was used in one study and SonoVue was used in the others, at a dose of 0.2–2.4 ml. The CEUS mechanical index ranged from 0.05 to 0.20. All diagnoses were confirmed by pathological examination. And the percentage of patients with sccRCC ≤4 cm showing each CEUS characteristic is listed in Table 2. The reasons that the multivariate analysis could not be performed in the following analysis owning to few articles which could be incorporated into, and that the multiple CEUS features of the same lesion for sccRCC ≤4 cm could not be extracted, which we regret to.

**TABLE 1:** General characteristics of included studies

Author	Year	Geographic location	Language	Ethnicity	Gender(M/F)	Age (year)	Study design	sccRCC (n)	Length (cm)	Instrument	Contrast agent type	Dose (mL)	Mechanical index	Gold standard
Jiang	2010	China	English	Asian	73/16	56	Retrospective	60	≤4	Siemens	SonoVue	1.2	0.11–0.18	Pathology
Wu	2011	China	Chinese	Asian	45/25	52 ± 12	Retrospective	63	≤3	GE	SonoVue	2.4	0.08	Pathology
Lei	2012	China	Chinese	Asian	73/59	45.1 ± 10.5	Retrospective	95	≤3	GE, Siemens, Acuson sequoians	SonoVue	—	0.08‐0.10	Pathology
Oh	2014	Iksan, Korea	English	Iksan, Korea	35/14	61	Retrospective	33	≤4	—	SonoVue	—	—	Histopathology
Atri	2015	Canada	English	Canadian	56/35	62 ± 14	Retrospective	41	≤4	Sequoia	Definity	0.2	0.06	Histopathology
Lu	2015	China	English	Asian	115/74	47.3 ± 20.7	Retrospective	72	≤4	GE	SonoVue	1.2	<0.1	Histopathology
Li	2016	China	English	Asian	216/89	52.4 ± 12.7	Retrospective	70	≤3	Philips, GE, Genoa	SonoVue	1.2	0.05‐0.11	Pathology
Yuan	2016	China	English	Asian	42/30	35.9 ± 11.7	Retrospective	55	≤3	IU22, Philips	SonoVue	2.4	0.07‐0.20	Pathology
Zhang	2017	China	Chinese	Asian	55/40	54.91 ± 11.5050	Retrospective	59	≤3	Philips	SonoVue	1.5	—	Pathology
Li	2019	China	Chinese	Asian	41/19	51.25 ± 9.21	Retrospective	49	≤4	GE	SonoVue	—	—	Pathology

**TABLE 2:** Incidences of CEUS characteristics in patients with sccRCC ≤4 cm

CEUS characteristics	Total cases (*n*)	The cases present in sccRCC	The percentage
Heterogeneous enhancement	353	196	55.5%
Homogeneous enhancement	353	157	44.5%
Hyperenhancement	200	289	69.2%
Iso‐enhancement	289	72	24.9%
Hypo‐enhancement	289	17	5.9%
Hyper‐iso‐enhancement	289	272	94.1%
Fast‐in	391	342	87.5%
Slow‐in	391	37	9.5%
Iso‐in	391	12	3.0%
Present of the pseudocapsule	281	149	53.0%
Absent of the pseudocapsule	281	132	47.0%
Fast‐out	424	255	60.1%
Slow‐out	424	150	35.4%
Iso‐out	424	19	4.5%

**FIGURE 2:**
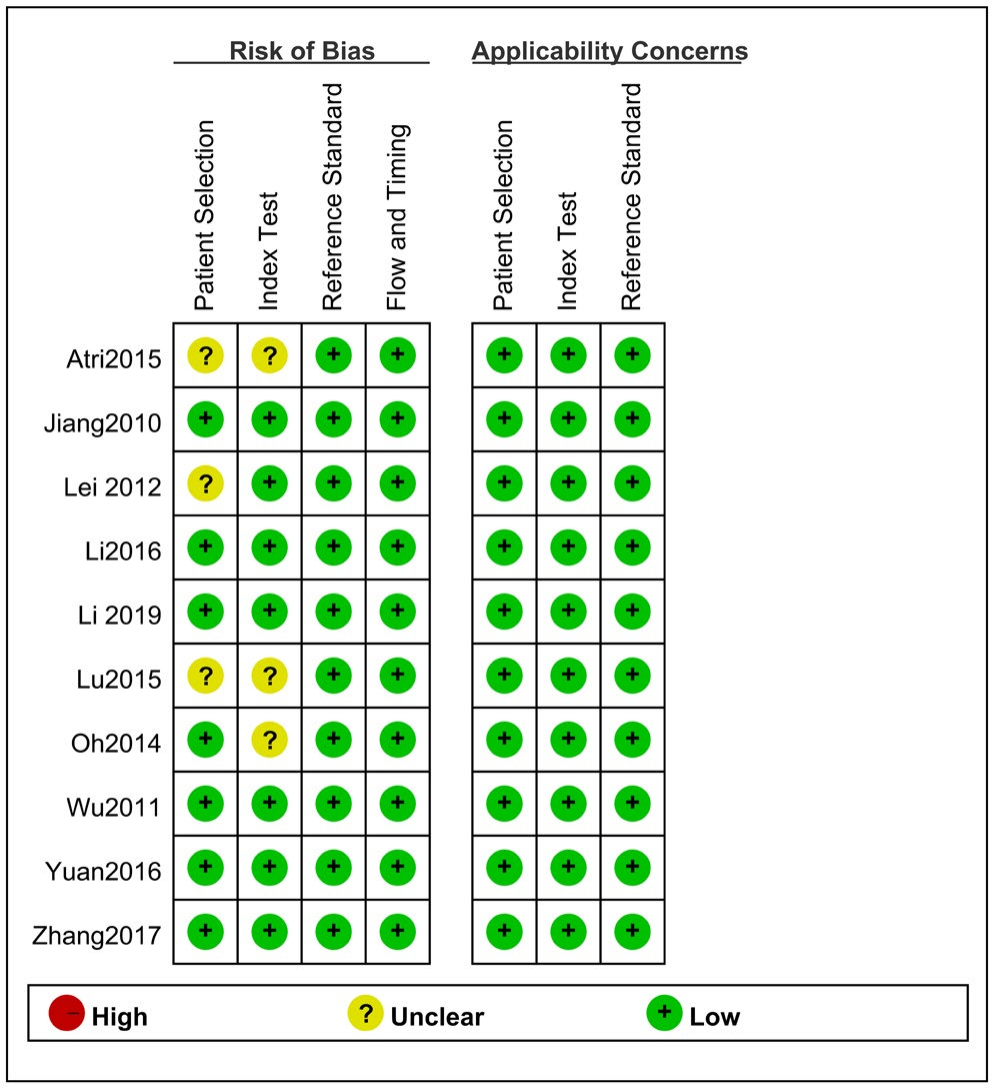
Overall risk of bias for each domain for each study

The assessment of the methodological quality of the included articles using the QUADAS‐2 tool is depicted in Figure 2. The unclear risk of bias in the papers by Oh et al., Lei et al., and Atri et al.^2^, ^11^, ^12^ was associated with “patient selection,” and “Index Test” also contributed to the unclear concerns in the studies by Oh et al.,^11^ Atri et al.,^2^ and Lu et al.^13^

The inappropriate exclusion of patients and lack of reviewer blinding to the final pathologic diagnosis were the main contributors to overestimation of diagnostic accuracy. The remaining QUADAS‐2 domains were all considered to have low risk of bias and low concern for all studies.

### 3.2 Synthesis of results

#### 3.2.1 CEUS characteristics with AUC ≥0.70 in patients with sccRCC ≤4 cm

The sensitivity (*p* < 0.05, *I* ^2^ = 82.57%) and specificity (*p* < 0.05, *I* ^2^ = 90.14%) of heterogeneous enhancement showed significant heterogeneity, with a combined sensitivity of 0.67 (95% CI: 0.51–0.79) and a combined specificity of 0.75 (95% CI: 0.45–0.92). The SROC with pooled AUC was 0.75 (95% CI: 0.71–0.78). The summary PLR and NLR were 2.7 (95% CI: 1.1–6.3) and 0.44 (95% CI: 0.32–0.62), respectively, the DOR was 6 (95% CI: 2–17), and the pooled OR was 5.882 (95% CI: 2.168–15.968).

For hyperenhancement, the sensitivity (*p* < 0.05, *I^2^* = 81.01%) and specificity (*p* < 0.05, *I^2^* = 84.30%) also showed significant heterogeneity, with a combined sensitivity of 0.70 (95% CI: 0.49–0.84) and a combined specificity of 0.67 (95% CI: 0.45–0.84). The SROC with pooled AUC was 0.74 (95% CI: 0.70–0.77). The summary PLR and NLR were 2.1 (95% CI: 1.4–3.3) and 0.45 (95% CI: 0.30–0.69), respectively, the DOR was 5 (95% CI: 3–9), and the pooled OR was 5.118 (95% CI: 2.758–9.496).

The sensitivity of fast‐in contrast agent showed slight heterogeneity (*p* < 0.05, *I* = 71.70%) and the specificity showed significant heterogeneity (*p* < 0.05, *I* = 3.12%). The combined sensitivity was 0.89 (95% CI: 0.81–0.94) and the combined specificity was 0.42 (95% CI: 0.18–0.69). The SROC with pooled AUC was 0.84 (95% CI: 0.80–0.87). The summary PLR and NLR were 1.5 (95% CI: 0.9–2.5) and 0.27 (95% CI: 0.11–0.63), respectively, the DOR was 6 (95% CI: 2–21), and the pooled OR was 5.991 (95% CI: 1.711–20.978).

#### 3.2.2 CEUS characteristics with AUC ≤0.70 in patients with sccRCC ≤4 cm

The sensitivity (*p* < 0.05, *I* ^2^ = 95.27%) and specificity (*p* < 0.05, *I* ^2^ = 85.73%) of the presence of a pseudocapsule both showed significant heterogeneity, with a combined sensitivity of 0.54 (95% CI: 0.26–0.79) and a combined specificity of 0.63 (95% CI: 0.46–0.77). The SROC with pooled AUC was 0.63 (95% CI: 0.59–0.67). For the absence of a pseudocapsule, the sensitivity (*p* < 0.05, *I* ^2^ = 95.27%) and specificity (*p* < 0.05, *I* ^2^ = 85.73%) also showed significant heterogeneity, with a combined sensitivity of 0.46 (95% CI: 0.21–0.74) and a combined specificity of 0.37 (95% CI: 0.23–0.54). The SROC with pooled AUC was 0.37 (95% CI: 0.33–0.41). For fast‐out contrast agent, the sensitivity showed significant heterogeneity (*p* < 0.05, *I* ^2^ = 93.13%) and the specificity showed mild heterogeneity (*p* < 0.05, *I* ^2^ = 47.37%), and the combined sensitivity was 0.62 (95% CI: 0.42–0.78) and the combined specificity was 0.28 (95% CI: 0.18–0.41). The SROC with pooled AUC was 0.34 (95% CI: 0.30–0.39). The sensitivity for homogeneous enhancement showed slight heterogeneity (*p* = 0.05, *I* ^2^ = 57.41%), the specificity showed significant heterogeneity (*p* = 0.05, *I* ^2^ = 84.39%), the combined sensitivity was 0.41 (95% CI: 0.33–0.50), and the combined specificity was 0.19 (95% CI: 0.05–0.54). The SROC with pooled AUC was 0.35 (95% CI: 0.731–0.39). The summary PLR, NLR, and DOR were all low for all the above features.

#### 3.2.3 CEUS characteristics with sensitivity >90% in patients with sccRCC ≤4 cm

Hyper‐iso‐enhancement showed high sensitivity (0.98, 95% CI: 0.86–1.00) and poor specificity (0.38, 95% CI: 0.26–0.53). The SROC with pooled AUC was 0.56 (95% CI: 0.52–0.60) for hyper‐iso‐enhancement (≤0.70). The sensitivity (*p* = 0.01, *I* ^2^ = 74.05%) showed slight heterogeneity and the specificity (*p* = 0.39, *I* ^2^ = 0.59%) showed low heterogeneity. The DOR was 5 (95% CI: 3–9), the pooled OR was 10.522 (95% CI: 5.106–21.682), and the summary PLR and NLR were low.

**FIGURE 3:**
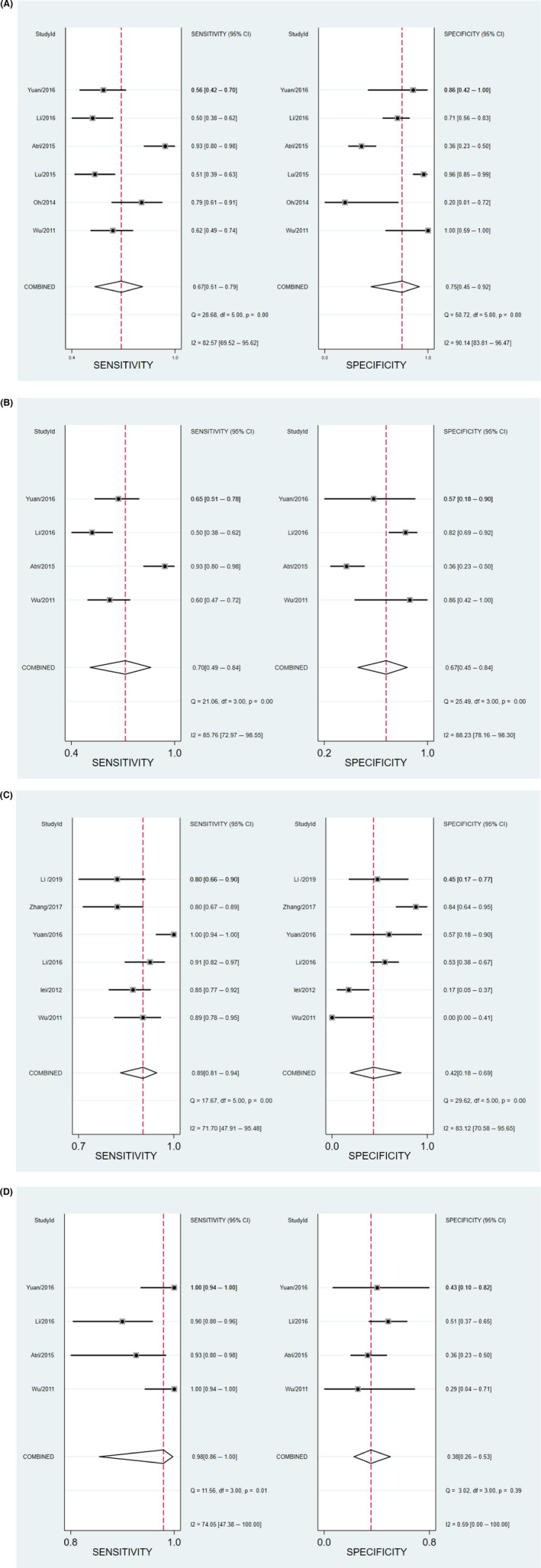
Forest plot of pooled sensitivity and specificity of CEUS characteristics including (A) heterogeneous enhancement, (B) hyperenhancement, (C) fast‐in of contrast agent, and (D) hyper‐iso‐enhancement in patients with sccRCC ≤4 cm

**FIGURE 4:**
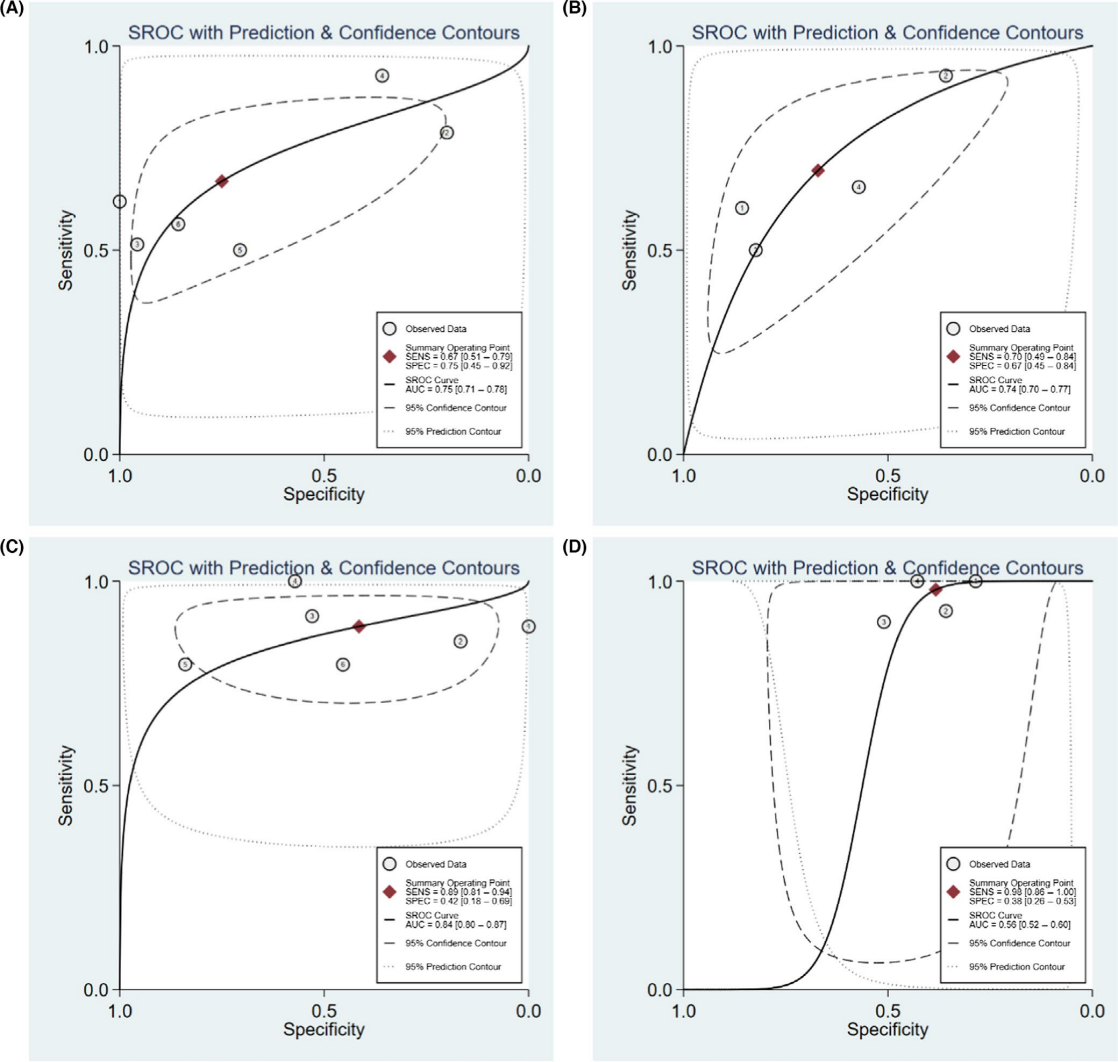
The SROC curves of CEUS characteristics including (A) heterogeneous enhancement, (B) hyperenhancement, (C) fast‐in of contrast agent, and (D) hyper‐iso‐enhancement in patients with sccRCC ≤4 cm

The forest plot of pooled sensitivity and specificity of CEUS characteristics including heterogeneous enhancement, hyperenhancement, fast‐in of contrast agent, and hyper‐iso‐enhancement, respectively, in patients with sccRCC ≤4 cm is shown in Figure 3. SROC curves of CEUS characteristics including heterogeneous enhancement, hyperenhancement, fast‐in of contrast agent, and hyper‐iso‐enhancement, respectively, in patients with sccRCC ≤4 cm are shown in Figure 4.

### 3.3 Subgroup analyses according to year of publication, language, ethnicity, lesion length, and contrast agent type

Subgroup analyses were conducted based on the year of publication, language, ethnicity, lesion length, and contrast agent type to investigate potential sources of heterogeneity. There was no significant heterogeneity for hyperenhancement in any of the aspects. For hyper‐iso‐enhancement, slight heterogeneity (*p* = 0.101, *I* ^2^ = 62.8%) was observed in two studies published in 2016, and slight heterogeneity (*p* = 0.081, *I* ^2^ = 67.2%) was also found in two studies that focused on lesions within 4 cm. Regarding fast‐in contrast agent, slight or significant heterogeneity was seen in all aspects. For fast‐out contrast agent, slight heterogeneity (*p* = 0.016, *I* ^2^ = 64.1%) was found in six studies in Asian patients, slight heterogeneity (*p* = 0.016, *I* ^2^ = 64.1%) was observed in six studies using SonoVue, and significant heterogeneity (*p* = 0.006, *I* ^2^ = 75.7%) was detected in four studies focusing on lesions within 3 cm. Regarding the presence/absence of a pseudocapsule, there was significant heterogeneity in two studies published in 2016, three studies published in English, three studies in Asian populations, two studies of lesions within 3 cm, and six studies using SonoVue. For heterogeneous enhancement, slight heterogeneity was observed in five studies in English, four studies in Asians, two studies focused on lesions within 3 cm, and five studies using SonoVue. For homogeneous enhancement, there was slight heterogeneity in four studies in English (*p* = 0.023, *I* ^2^ = 68.5%), four studies in Asian populations (*p* = 0.023, *I* ^2^ = 68.5%, two studies focusing on lesions within 3 cm (*p* = 0.120, *I* ^2^ = 58.7%), three studies focusing on lesions within 4 cm (*p* = 0.070, *I* ^2^ = 62.4%), and five studies using SonoVue (*p* = 0.024, *I* ^2^ = 64.3%). The above results identified these factors as potential sources of heterogeneity.

### 3.4 Sensitivity analysis and publication bias

We carried out sensitivity analysis for each CEUS characteristic. The results indicated that, for hyperenhancement and hyper‐iso‐enhancement, removal of the study by Atri et al.^2^ divorced the mean odds ratio (OR) value from the 95% CI, suggesting that this article may have had a major influence on the OR. Removal of each study in turn had little effect on the mean ORs for the remaining CEUS characteristics, suggesting that the overall pooled results were stable. In addition, Begg's and Egger's tests and funnel plots showed no publication bias in the included studies.

## 4 DISCUSSION

The imaging findings of sccRCC are largely determined by the lesion's pathological features and the sizes. A size of 4 cm is considered as a cut‐off point for sccRCC, with differences in blood vessels, degree of compression of the adjacent normal cortex, and number of arteriovenous fistulas between sccRCC lesions >4 cm and ≤4 cm. Nevertheless, the guidelines do not point out the definitive definition about CEUS features for sccRCC ≤4 cm.^5^ And it is rarely reported in the field of the CEUS characteristics in sccRCC ≤4 cm now. For the first time, we conducted a comprehensive online database search to explore the CEUS findings in sccRCC ≤4 cm.

### 4.1 CEUS characteristics with high diagnostic ability or sensitivity for sccRCC ≤4 cm

The CEUS characteristics of sccRCC depend on its vascular pathological features. Compared with normal blood vessels, neovascularization in renal tumors is characterized by an increased number of blood vessels with enlarged diameter, compression, displacement, and a disorganized structure, with irregular secondary branches, incomplete basement membrane, and abnormal formation of sinuses and arteriovenous fistulas.^14^

**FIGURE 5:**
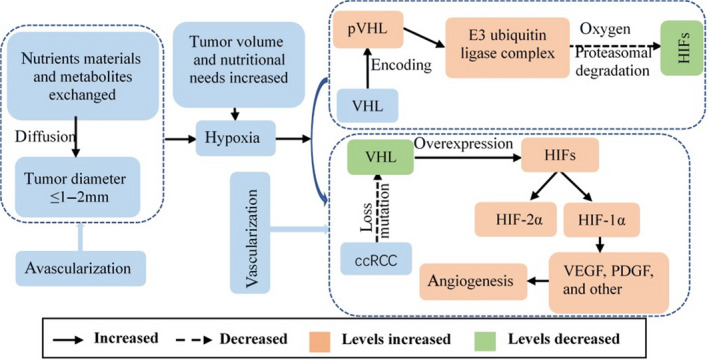
Avascular and vascular growth phases of ccRCC, and mechanism of angiogenesis under a hypoxic state, involving *VHL* gene loss or mutation

The mechanism of angiogenesis in ccRCC is shown in Figure 5. Most renal tumors are solid tumors, and their growth can be considered as either avascular or vascular. In the avascularization phase, tumor aggregates form and sufficient nutrients and metabolites can be exchanged and transported between the tumor cells and surrounding tissues by diffusion, as long as the tumor diameter is ≤1–2 mm. However, as the tumor volume increases, its nutritional needs can no longer be met through simple diffusion,^10^ and the tumor enters the vascularized growth phase. In the absence of new blood vessel growth, the tumor tissues remain dormant or degenerate,^15^ and the tumor cells become hypoxic. The von Hippel–Lindau gene (*VHL*) encodes an oxygen‐sensing mediator protein (pVHL) in the hypoxia signaling pathway.^16^ pVHL is a crucial component of the E3 ubiquitin ligase complex, which is necessary for exposing the family of hypoxia‐inducible transcription factors (HIFs) for proteasomal degradation in the presence of oxygen.^16^, ^17^ ccRCC is characterized by genetic loss or mutation of *VHL*,^16^ resulting in metabolic dysregulation, heightened angiogenesis, intratumoral heterogeneity, and deleterious tumor microenvironment crosstalk.^18^ Under a hypoxic state, ccRCC cells overexpress HIF‐related pro‐angiogenesis (HIF‐1α and HIF‐2α) and glycolysis target genes, which in turn stimulate the production of related growth factors such as vascular endothelial growth factor, platelet‐derived growth factor, and other growth factors, leading to increased tumor angiogenesis, which promote tumor proliferation, growth, and metastasis, and affect the prognosis.^19^

**FIGURE 6:**
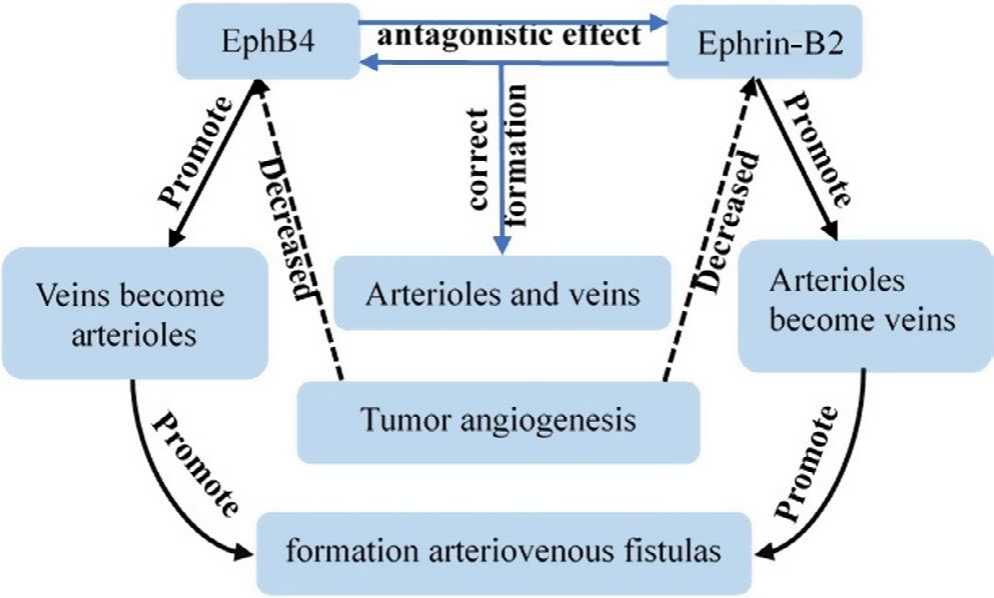
Antagonistic effects of EphB4 and ephrin‐B2 guide the differentiation of arterioles and veins, and disruption of this relationship in ccRCC may result in the formation of arteriovenous fistulas

EphB4/ephrin‐B2 is widely involved in physiological and pathological angiogenesis during embryonic and postnatal development, and is considered to be molecular markers of arteriovenous development.^20^ There is an antagonistic effect between ephrin‐B2 and EphB4, which guides the orientation and spatial combination of endothelial cells to ensure the correct formation of arteriovenous vessels and the boundaries between arterial and venous capillaries. Once the blood vessels have been formed, the blood supply involves perfusion, and endogenous cell signaling and perfusion may conversely affect the expression of ephrin‐B2 during differentiation of the original capillary plexus into arteries or veins.^20^ A change in perfusion direction may cause existing arterioles to become veins, with decreased ephrin‐B2 expression, and vice versa for veins, possibly also resulting in the abnormal formation of sinuses and arteriovenous fistulas (Figure 6).

The sccRCC characteristics of hyperenhancement, heterogeneous enhancement, and fast‐in contrast agent depend on the structural and functional abnormalities of the tumor blood vessels. The current analysis indicated that hyperenhancement, fast‐in contrast agent, and heterogeneous enhancement had high diagnostic ability (AUC ≥0.70) for sccRCC lesions ≤4 cm, with sensitivities of 70%, 89%, and 67% and specificities of 67%, 42%, and 75%, respectively. These results were in accordance with previous studies that reported “hyperenhancement,” “fast‐in,” and a “peritumoral hyperenhanced rim (pseudocapsule)” as CEUS characteristics of ccRCC.^21^ Sun et al. analyzed the qualitative characteristics of CEUS and identified “fast to peak + high peak intensity” as the main criterion, while the addition of “heterogeneous enhancement” and “early wash‐in” maximized the diagnostic accuracy for ccRCC at 91.4%.^22^ We was also analyzed the combination of hyperenhancement and iso‐enhancement, and showed that this combined index demonstrated high sensitivity (98%) but low specificity (38%), and the AUC (0.56) indicated that this combination had a poor detection ability for sccRCCs ≤4 cm. sccRCC is a highly vascularized malignant tumor with extensive angiogenesis,^23^ and the high number and large diameter of the blood vessels usually results in hyperenhancement compared with the adjacent normal renal cortex.^21^ In addition, the contrast agent enters the lesion rapidly through the arteriovenous fistulas in the arterial phase, resulting in fast‐in contrast agent characteristics. These results agree with those of Chen et al.^24^ The tumor grows rapidly during the vascularized phase, and ischemic necrosis occurs when the new blood vessels cannot provide enough nutrients to meet the tumor's growth needs, resulting in heterogeneous enhancement on CEUS. However, the incidence rate (55.5%) of heterogeneous enhancement in the present meta‐analysis was lower than that in some previous studies. This may be because of the size of the tumor; increasing tumor size is associated with more frequent hemorrhage and necrosis, and heterogeneous enhancement due to necrosis may thus be more common in tumors >4 cm, which were excluded from our study. CEUS characteristics may also depend on the clinical TNM staging, with experts noting that hyperenhancement and heterogeneous enhancement were more likely in stage pT3 and pT4 tumors. However, we did not explore this correlation between TNM stage and CEUS characteristics in the current study.

### 4.2 CEUS characteristics with poor diagnostic ability for sccRCC ≤4 cm

The presence of a pseudocapsule is related to tumor size, as confirmed by Atri et al.^2^ and Jiang et al.^25^ Dai et al.^26^ also indicated that fewer pseudocapsule signs existed in tumors <2 cm and tumors >6 cm. A tumor pseudocapsule present in CEUS as a rim‐like enhancement around the tumor, reflecting compression of the adjacent normal parenchyma by the growing tumor, resulting in ischemia and necrosis of the compressed tissue and subsequent deposition of fibrous tissue. The presence of a pseudocapsule in this review showed medium specificity (63%) and sensitivity (54%, similar to the 56.7% (34/60) reported by Jiang et al.^25^ in ccRCCs ≤3 cm). However, this figure was significantly lower than in some other studies, and Xu et al.^27^ reported the presence of rim‐like enhancement in 79.6% (74/93) of RCCs. This difference may be because of the bigger sample, and because only masses ≤4 cm were included in the current study. In addition, experts have noted that the probability of detecting a pseudocapsule depend on the differentiation level. Tumors with a high nuclear grade tended to show invasion of the tumoral pseudocapsule, resulting in a low detection rate, while the presence of a pseudocapsule and homogeneous enhancement were more commonly seen in stage pT1 tumors. The current study did not analyze the impact of differentiation degree and TNM stage on the presence of a pseudocapsule and homogeneous enhancement, and further studies are needed to explore these relationships.

Most studies indicated that ccRCC demonstrated fast‐out contrast agent properties. However, the current analysis suggested that fast‐out contrast agent had a poor detection ability, with a sensitivity of only 0.62, low specificity, and poor overall diagnostic ability (AUC 0.34). The apparent discrepancy between these results and those of others studies may be related to the intra‐tumoral heterogeneity. The fast‐out loss of contrast agent occurs via arteriovenous fistulas or by normal physiological reflux of renal veins. However, intra‐tumoral heterogeneity derived from clonal and sub‐clonal tumor cells may affect the differentiation degree and malignancy level of the lesion,^28^ with greater malignancy associated with greater invasion of the surrounding tissues. This may in turn affect the normal physiological reflux of the renal veins, leading to differences in wash out of the contrast agent in some lesions. Moreover, one quantitative study noted that the quantitative features for the fad of contrast agent remained controversy.^21^

## 5 LIMITATIONS

This study has several limitations. First, we did not include any gray literature or unpublished studies, because these were usually unavailable. Second, most of the included studies were from China and most of the study populations were Asian, with very few international studies including non‐Asian populations. This may have affected the results, and, further studies are needed to confirm the validity of the results in different populations. Third, the study data were from different regions and affiliations, and the CEUS images were evaluated by clinicians with different experiences, potentially affecting the diagnostic results. Finally, the number of included studies was small, making it impossible to perform some subgroup meta‐regression and sensitivity and specificity analyses, thus highlighting the need for more studies in the future. More research is also needed to examine the quantitative parameters in sccRCC ≤4 cm, and to explore the qualitative and quantitative parameters in lesions >4 cm. Due to the inability to extract multiple CEUS features from the same lesion, we were unable to analyze the combined diagnostic values of multiple features for sccRCC lesions ≤4 cm, and we aim to address this issue in further studies.

Hyperenhancement, fast‐in contrast agent, and heterogeneous enhancement may have high diagnostic ability for sccRCC lesions ≤4 cm. Hyperenhancement and iso‐enhancement showed high sensitivity for diagnosing sccRCC ≤4 cm, suggesting that the combined index may be particularly useful for detecting these lesions. In contrast, presence of a pseudocapsule and fast‐out of contrast agent might not aid the diagnosis of sccRCC lesions ≤4 cm.

## CONFLICT OF INTEREST

The authors declare that they have no conflict of interest.

## INFORMED CONSENT

Institutional Review Board approval and informed consent from the patient are not required because this is a systematic review of published literature.

## Data Availability

The data that support the findings of this study are openly available in Open database.
